# Modeling Neurological Disease by Rapid Conversion of Human Urine Cells into Functional Neurons

**DOI:** 10.1155/2016/2452985

**Published:** 2015-12-07

**Authors:** Shu-Zhen Zhang, Li-Xiang Ma, Wen-Jing Qian, Hong-Fu Li, Zhong-Feng Wang, Hong-Xia Wang, Zhi-Ying Wu

**Affiliations:** ^1^Department of Neurology, Institute of Neurology, Huashan Hospital, Institutes of Brain Science and State Key Laboratory of Medical Neurobiology, Shanghai Medical College, Fudan University, Shanghai 200040, China; ^2^Department of Neurology, Research Center of Neurology in Second Affiliated Hospital, and the Collaborative Innovation Center for Brain Science, Zhejiang University School of Medicine, Hangzhou 310009, China; ^3^Department of Anatomy, Histology & Embryology, Shanghai Medical College, Fudan University, Shanghai 200030, China; ^4^Institutes of Brain Science, Institute of Neurobiology and State Key Laboratory of Medical Neurobiology, Fudan University, Shanghai 200030, China

## Abstract

Somatic cells can be directly converted into functional neurons by ectopic expression of defined factors and/or microRNAs. Since the first report of conversion mouse embryonic fibroblasts into functional neurons, the postnatal mouse, and human fibroblasts, astroglia, hepatocytes, and pericyte-derived cells have been converted into functional dopaminergic and motor neurons both *in vitro* and *in vivo*. However, it is invasive to get all these materials. In the current study, we provide a noninvasive approach to obtain directly reprogrammed functional neurons by overexpression of the transcription factors Ascl1, Brn2, NeuroD, c-Myc, and Myt1l in human urine cells. These induced neuronal (iN) cells could express multiple neuron-specific proteins and generate action potentials. Moreover, urine cells from Wilson's disease (WD) patient could also be directly converted into neurons. In conclusion, generation of iN cells from nonneural lineages is a feasible and befitting approach for neurological disease modeling.

## 1. Introduction

Reprogramming techniques have been used to generate induced pluripotent stem cells (iPSCs) from human fibroblasts [1]. Patient-derived iPSCs differentiating into mature cells could be useful tools for disease modeling and cell-based therapy [2]. Patients with age-related macular degeneration have benefited from the iPSCs-derived retinal pigmented epithelium cell treatment [3]. However, the lengthy procedures of the iPSCs induction and target cells differentiation [4], in addition to the tumorigenic properties [1], restricted the use of iPSCs.

Direct reprogramming is another option to generate patient-specific cells. Reprogrammed cells do not pass through the pluripotent state and the entire conversion procedure takes less than two months. Therefore, reprogrammed cells may serve as potential alternative tools for disease modeling and cell-based therapy. An important milestone in the field of cell lineage conversions was the discovery that the single factor MyoD was sufficient to convert fibroblasts to myoblasts [5]. Somatic cells have been successfully converted into myoblasts [6], chondrocytes [7], functional cardiomyocytes [8], functional hepatocyte-like cells [9], hepatic stem cells [10], multilineage blood progenitors [11], neural stem cells [12], functional neurons [13–15], even more specific neurons such as motor neurons [16], and dopaminergic neurons [17, 18].

The most commonly used somatic cells for direct conversion are fibroblasts [16]. The alternative materials are brown fat cells [6], placenta [7], hepatocytes [14], pericyte-derived cells [15], and astrocytes [18]. However, it is invasive to get these cells. Recently, several groups generated human iPSCs [19, 20] and neuron stem cells [21] from exfoliated renal epithelial cells present in urine, providing a noninvasive approach to obtain reprogramming cells. The isolation of urinary cells is simple, cost-effective, and universal. However, the direct conversion of urine cells to functional neurons has not been reported so far.

In the present study, we showed that human urine cells could be directly reprogrammed into functional neurons using a combination of Ascl1, Brn2, NeuroD, c-Myc, and Myt1l and various neurotrophic factors. These induced neuronal (iN) cells expressed multiple neuron-specific proteins and generated action potentials. Generation of iN cells from nonneural lineages could have important implications for neurological disease modeling and regenerative medicine.

## 2. Materials and Methods

### 2.1. Culture and Verification of Human Urinary Cells

The urinary cells were collected from healthy individuals and WD patients after obtaining informed consents. The exfoliated cells were then isolated using a previously established protocol with brief modifications [19]. Donors were asked to drink 300–500 mL water before urine collection. The vulva was wiped with iodine to keep sterile. Approximately 200 mL aseptic middle stream of the micturition was collected into a sterilized glass bottle. The urines were centrifuged at 1000 rpm for 15 min at room temperature immediately after collection. The upper liquid was discarded, and 2 mL renal epithelial cell growth medium (REGM) with growth factors (Lonza, cat. number CC-4127) was added to the bottom sediment. The cell-like organisms were seeded on one well of 24-well plate (Corning) at a density of about 0.5*∗*10^6^/cm^2^. Half of the medium was discarded and replaced by 1 mL of new REGM medium per day for 3 successive days. Small clones normally appeared at day 5 after planting. Usually one to five small colonies were observed per well. Cells exhibited short spindle morphologies when they reached confluence. These cells were subcultured or frozen for further usage. The study protocol was approved by the research ethics committee of Huashan Hospital.

### 2.2. Reprogramming of Urinary Cells

Five retroviruses (Lenti-EF1*α*-EGFP-TRE-ASCL1, Lenti-EF1*α*-EGFP-TRE-BRN2, Lenti-EF1*α*-EGFP-TRE-NEUROD, Lenti-EF1*α*-EGFP-TRE-MYT1L, and Lenti-EF1*α*-EGFP-TRE-C-MYC) were produced for the ectopic expression of human Ascl1, Brn2, NeuroD, c-Myc, and Myt1l, respectively. Retroviruses carrying these five transcriptional factors were produced in the 293T cell line. Viral supernatant was concentrated by Amicon Ultra Centrifugal Filters (Millipore) and filtered through 0.22 *μ*m cellulose acetate filter before being added to urine cells. Primary monolayer astrocyte cultures were established as described previously [22].

Twenty-four hours after the recovery, the five retroviruses were mixed at 1 : 1 : 1 : 1 : 1 and were added to 1*∗*10^5^ primary urinary cells at multiplicity of infection (MOI) of 7.5–10. The medium was changed to 1 : 1 mixture of EPi and N2 medium (EPi/N2, DMEM/F2, N2, and NEAA [Invitrogen]) supplied with 1 *μ*g/mL DOX for 2 days. Cells were harvested and plated onto astrocyte coated cover slips (1 × 10^4^/cm^2^) in N2 medium supplemented with DOX, Y21732 (10 *μ*M, Tocris), cAMP (1 *μ*M), Vit-C (200 *μ*M), 0.5% FBS (Gibco), and neurotrophic factors including NT-3, BDNF, GDNF, and IGF (10 ng/mL each, R&D systems). The culture medium was changed every other day until used.

### 2.3. RNA Isolation and Relative Quantitative PCR

Total RNA was extracted using MiniBEST Universal RNA Extraction Kit (TaKaRa). The reverse-transcription reaction was conducted using the PrimeScript RT Master Mix (TaKaRa). Primers used are the same as those reported previously [19]. SYBR Premix Ex Taq (TaKaRa) was used for the relative quantitative PCR reaction.

### 2.4. Electrophysiology

The electrophysiology experiment was performed according to the previously described protocol with some modifications [23]. Cells selected for electrophysiological recordings had neuron-like shapes with fine branching nerve projections. Action potentials were recorded with current-clamp whole cell configuration. The bath solution contained the following (in mM): 125 NaCl, 2.5 KCl, 25 NaHCO_3_, 1.25 NaH_2_PO_4_, 2 CaCl, 1 MgCl_2_, and 25 glucose. The pipette solution contained the following (in mM): 123 K-gluconate, 10 KCl, 1 MgCl_2_, 10 HEPES, 1 EGTA, 0.1 CaCl_2_, 1 K_2_ATP, 0.2 Na_4_GTP, and 4 glucose, pH adjusted to 7.2 with KOH. Membrane potentials were kept around −65 to −70 mV, and step currents were injected to elicit action potentials. For whole cell voltage-dependent current recordings, the same internal solution as aforementioned was used.

### 2.5. Immunofluorescence

Cells were fixed in 4% paraformaldehyde for 10 min and blocked by PBS solution containing 5% donkey serum and 0.2% Triton X-100 for 1 hour at room temperature. Primary antibodies were applied overnight and secondary antibodies were applied for 2 hours. The following antibodies were used: rabbit anti-Tuj1 (Covance, 1 : 1,000), mouse anti-Tuj1 (Covance, 1 : 1,000), mouse anti-MAP2 (Sigma, 1 : 500), rabbit anti-synaptophysin (Chemicon, 1 : 1,000), rabbit anti-v-Glu (Millipore, 1 : 2,000), and rabbit-anti-GABA (DSHB, 1 : 500). Alexa-488-, Alexa-594- and Cy5-conjugated secondary antibodies were obtained from Invitrogen. 4′,6-Diamidino-2-phenylindole (DAPI) was from Sigma (1 : 10,000).

### 2.6. Efficiency Calculation

The Tuj1-positive cells with thin processes at least three times longer than their cell bodies were counted. We selected 3 cover slips and averaged the number of iN cells. We then divided this number by the number of cells plated before infection to get the percentage of the starting population of cells that adopted neuron-like characteristics.

## 3. Results

**Figure 1:**
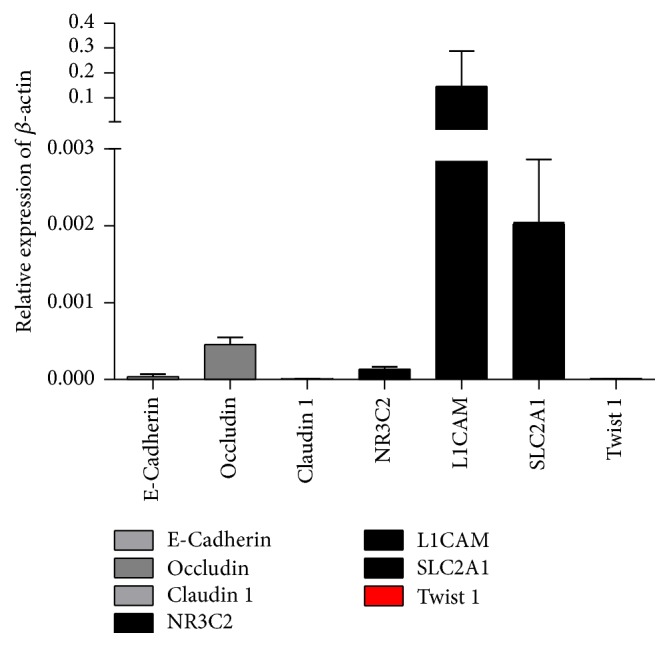
Cultured urine cells express renal epithelial and epithelial markers. The culture urine cells express renal epithelial cell markers NR3C2, L1CAM, and SLC2A1 at high level. Epithelial cell markers Occludin, Claudin 1, and E-cadherin were also expressed in those cells. The fibroblast marker Twist 1 was expressed at a very low level. *N* = 7.

To investigate whether human urinary cells could be directly converted into neurons, we collected urine samples from the volunteers with the informed consents. These cells expressed renal epithelial cell markers NR3C2, L1CAM, and SLC2A1 at higher levels and epithelial cell markers Occludin, Claudin 1, and E-cadherin at lower levels (Figure 1). The expression of Twist 1, a marker of fibroblast cell, was very low. This data indicated that in our culture systems most of the cells isolated from the urines were more similar to renal proximal tubular epithelial cells.

**Figure 2:**
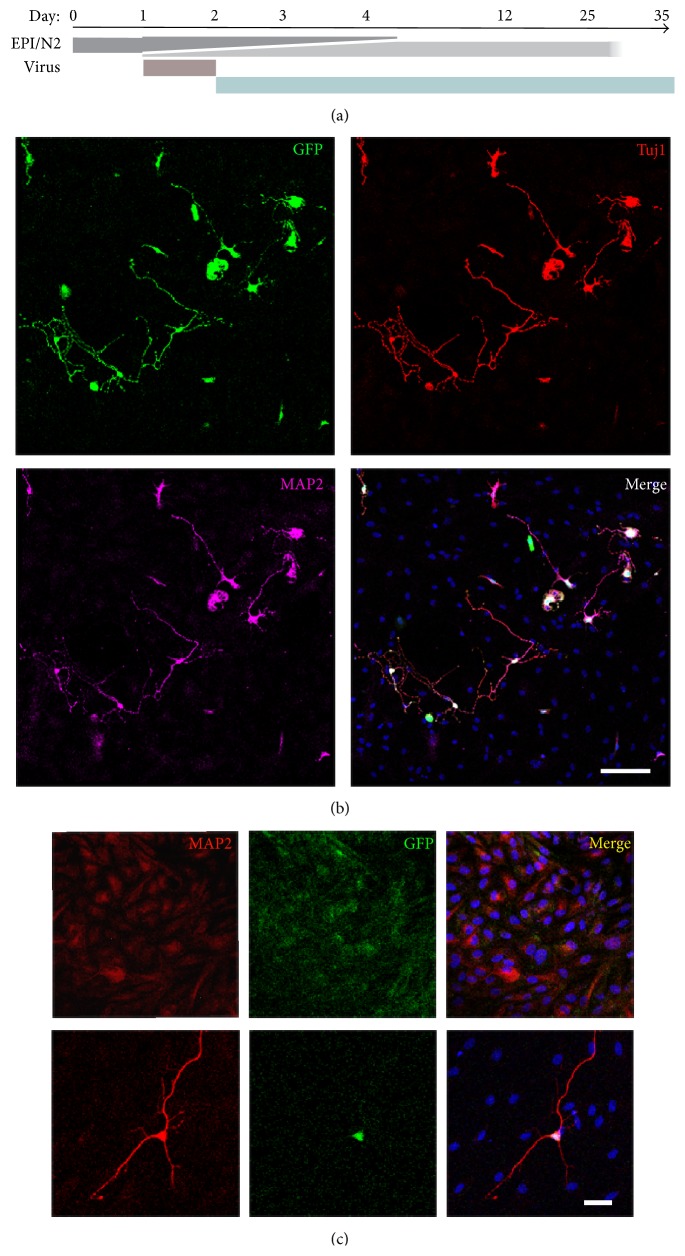
Generation of neurons from human urine cells. (a) Schematic protocol for conversion of urinary cells into neurons. (b) Twelve days after induction, urine derived-iN cells showed neuronal morphologies. And the urine-iN cells expressed both Tuj1 and MAP2. (c) Without the induction of DOX, the urine cells could not change into neuron cells (upper panel). In the presence of DOX, the DOX induced transcriptional factors (Ascl1, Brn2, NeuroD, c-Myc, and Myt1l) convert urine cells into neuron directly (lower panel). Scale bars, 50 *µ*m.

It was reported in 2011 that forced expression of neuronal lineage specific transcriptional factors Ascl1, Brn2, and NeuroD, in combination with Myt1l, could successfully convert human fibroblast into iN neurons [24]. So we tried these 4 transcriptional factors in the urinary cells. Unfortunately, these four factors could only convert urinary cells into neuron-like cells which died 4~6 days later, although we changed induction and culture condition. Then we tried the protooncogene Myc, which enhanced the efficiency of iPSC generation [25], in combination with the aforementioned 4 factors. We also changed the culture medium from N3 medium [24] to N2 medium which was used for the induction and maintenance of neurons induced from ES cells or iPSCs. To facilitate the survival of the neurons, Y-27632-dihydrochloride and FBS were added to the medium. Finally, urine cells were successfully converted into mature neurons. The conversion procedure was simplified as shown in Figure 2(a).

For the first 4 days, the cells showed epithelial-like morphology and sustained proliferation. From day 4, cells began to change their shape. We analyzed the expression of the transcriptional factors on day 4 by observing the GFP protein expression. We found that almost all the cells expressed GFP, but we could not know which cells expressed the 5 factors at the same time. On day 5, about 30% of the cells elongated and became long spindle cells. Some grew dendrite-like structures. Unfortunately, only a small percentage of these cells could be converted into neurons. Most of these cells began to die at almost the same time. So, we changed the medium every day from day 6, to remove the dead cells and retain the survival of the healthy cells. Cells which were successfully converted into neurons grew long processes and exhibited neuron-like morphology. And these cells could be labeled by neuron lineage marker Tuj1 (Supplementary Figure 1 in Supplementary Material available online at http://dx.doi.org/10.1155/2016/2452985).

Two weeks after infection with the aforementioned factors, the induced neuron cells showed neuronal morphologies and were labeled with neuronal antibodies Tuj1 and MAP2 (Figures 2(b) and 2(c)) in the DOX induced group but not in the group without DOX induction (Figure 2(c)). About 1.55% ± 0.01% (two independent experiments) of the initiated cells were converted into Tuj1-positive cells.

**Figure 3:**
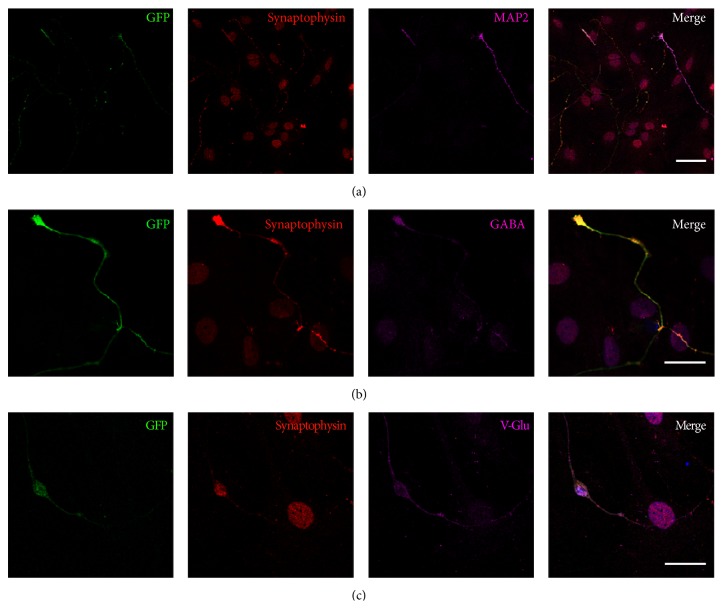
The iN cells expressed mature neuron marker synaptophysin, GABA, or v-Glu. (a) Twenty-four days after DOX treatment, the urine-iN cells expressed synaptophysin. (b) Immunofluorescent study revealed GABA positive iN cells. (c) Immunofluorescent study revealed that iN cells were positive for v-Glu. Scale bars, 50 *µ*m (a) and 20 *µ*m (b, c).

Immunofluorescence analysis showed that the iN cell expressed the mature neuron marker synaptophysin (Figure 3(a)) and there were GABAergic (Figure 3(b)) and glutamatergic (Figure 3(c)) neurons in the iN cells on day 25.

**Figure 4:**
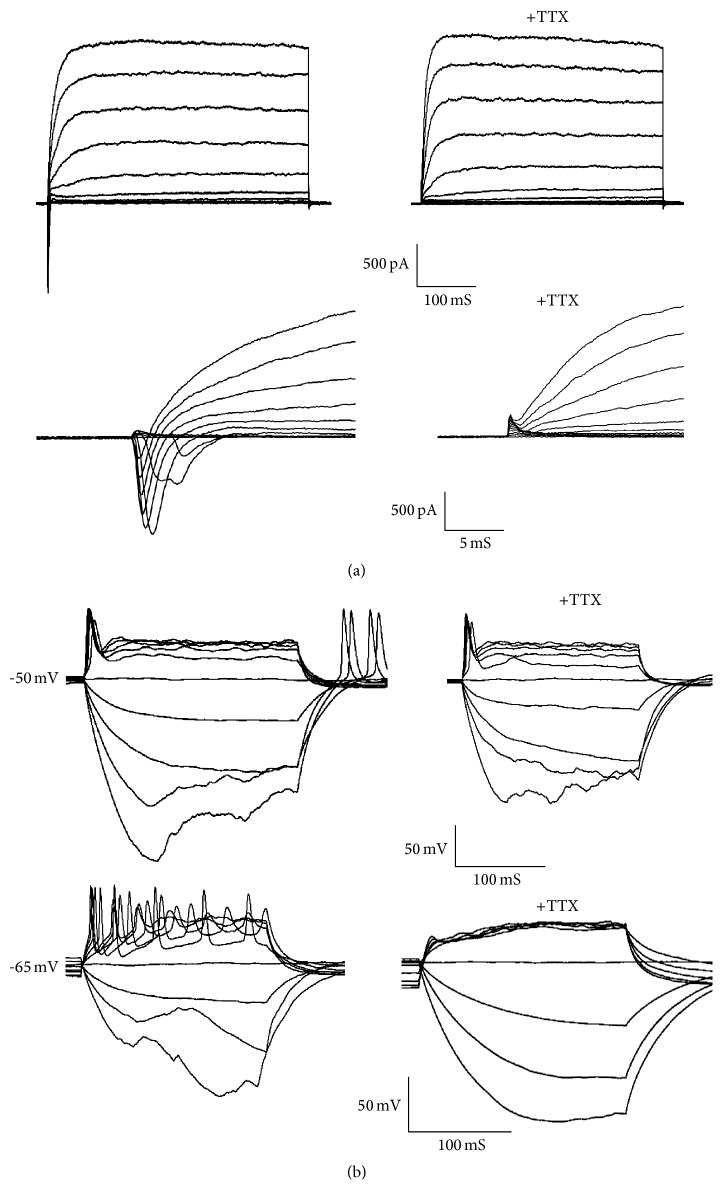
Membrane properties of the urine-iN cells. Whole cell recording was conducted on urine-iN cells identified by differential interference contrast microscopy. (a) Representative traces of membrane currents. Fast-activating and inactivating Na^+^ currents were prominent in all the iN cells. The Na^+^ currents could be blocked by tetrodotoxin (TTX). (b) Representative traces of action potentials in response to step current injections 35 days after induction. Membrane potential was maintained at approximately −52 mV. And the action potentials could be blocked by TTX treatment.

Though it expressed synaptic vesicle protein synaptophysin (Figure 3(a)) and fast-activating and inactivating inward Na^+^ currents as well as outward K^+^ currents could be induced (Figure 4(a)) on day 24, these cells remained functionally immature as revealed by their inability to generate action potentials (Figure 4(b), upper panel). The mean membrane capacity is 15.21 ± 2.73 pF (mean ± s.e.m., *n* = 33). The average resting membrane potential of iN cells was −44.89 ± 2.45 mV (mean ± s.e.m., *n* = 9). After extended culture periods to 5 weeks, the average resting membrane potential of iN cells is −49.50 ± 2.37 mV (mean ± s.e.m., *n* = 14), and we could detect induced action potentials which could be blocked by the TTX treatment (Figure 4(b), lower panel) in 21.4% (*n* = 14) of the iN cells.

**Figure 5:**
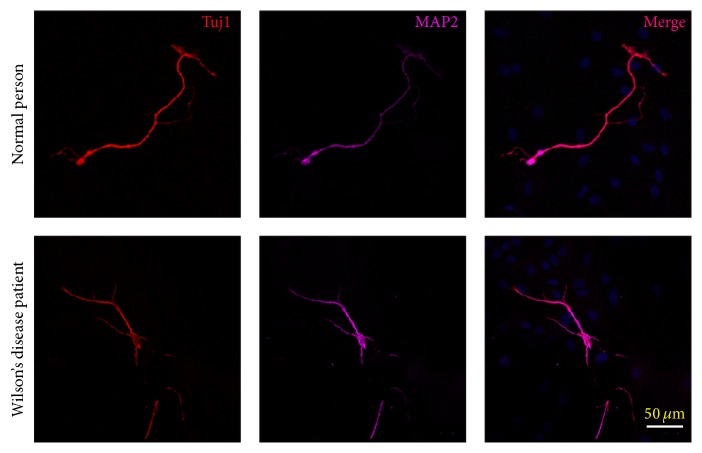
Urine cells from patients could be converted into neuron. Twelve days after doxycycline treatment, the urine from both normal individuals and WD patients could be converted into neurons as shown by the expression of Tuj1 and MAP2. Scale bars, 50 *µ*m.

To further verify this approach of direct conversion, we collected urinary cells from WD patients. Using the five factors mentioned above, we converted urine cells from both normal individuals and WD patients into neurons by day 12 (Figure 5).

## 4. Discussion

In this study, we found that a defined set of transcription factors (Ascl1, Brn2, NeuroD, c-Myc, and Myt1l) could directly reprogramme human urine cells into neuronal cells. To our knowledge, this is the first report concerning the direct conversion of human urine cells into functional neurons. These obtained iN cells could express multiple neuron-specific proteins and generate action potentials.

For decades, the paradigm of drug discovery and development as well as disease modeling had relied on immortalized cell lines and animal models of human diseases. However, candidate compounds for treating central nervous system defects failed in clinical trials in over 90% of cases due to poor targeting, lack of efficacy, and unacceptable side effects [26]. The last 10 years has witnessed the breakthrough discovery in bioscience. The generation of human iPSCs [1] has provided a platform for drug research and disease modeling for safety and efficacy. The direct reprogramming technique [13] has provided another* in vitro* drug testing platform for compound screening and exploring of the mysteries of various diseases. Researchers now have the opportunity to study human diseases in living, developing neural cells that carry the disease-specific genetic variants.

Until now, fibroblasts are the most commonly used somatic cells for direct reprogramming [8–13, 16, 17]. Besides, brown fat cells [6], placenta [7], hepatocytes [14], pericyte-derived cells [15], and astrocytes [18] are also used. The problem is that it is invasive to get them. Therefore it is hard to persuade patients to donate. The renal epithelial cells present in urine are idealized materials. The isolation of urinary cells is simple, cost-effective, and universal. The method for the collection and culture of urinary cells has also been established [20].

By forced expression of 5 transcription factors, Ascl1, Brn2, Myt1l, NeuroD, and c-Myc, we could generate functional neurons from human urine cells. The iN cells showed neuronal morphologies and were labeled with neuronal antibodies Tuj1 and MAP2. Most of the iN cells were glutamatergic neurons, which are the same as the data reported previously [24]. We could also observe the expression of synaptic vesicle protein synaptophysin and fast-activating and inactivating inward Na^+^ currents as well as outward K^+^ currents. Our results showed that neurons could be successfully reprogrammed from urine cells of healthy individuals.

Then we tried this method in WD patients' urinary cells. As shown in Figure 5, urinary cells from both healthy individuals and WD patients could be converted into neurons. Previous report has demonstrated that the direct reprogramming astrocytes from skin fibroblasts of amyotrophic lateral sclerosis showed similar toxicity towards motor neurons as astrocytes from autopsies of patients [27]. This means that this method could be used to establish cell models of neurological diseases, such as WD.

In 2012, Kim et al. reviewed the common feature of the direct conversion; first, the conversion process seems to be very rapid; second, the functional maturation of the iN cells seems to take several weeks; third, the efficiency of hiN cells generation is much lower in adult human cells than mouse adult cells; last, human iN cells derived from embryonic or neonatal human cells functionally and physiologically mature much faster than adult cell derived human iNs [28]. The reprogramming procedure of the urinary cells fits all the above features. It takes 5 days to see the cells which have neuronal morphology. Only about more than 1% of the urinary cells became neuron cells. And it takes 5 weeks to induce action potential in about only 21% of the iN cells. The most significant progress of the procedure is that the urinary cells are in endless supply, although the protooncogene c-Myc was used in this process.

## 5. Conclusion

This study demonstrates that exfoliated cells in urine could be efficiently converted into functional neurons. And we provide a new way for preclinical studies and disease modeling of neurological diseases.

## Supplementary Material

In order to convert urine cells into functional neurons, five retroviruses carrying Ascl1, Brn2, NeuroD, c-Myc, and Myt1l were used. Twenty-four hours after the recovery, the five retroviruses were added to primary urinary cells for one day. The medium was changed to EPi/N2 medium supplied with 1 µg/ml DOX the second day and last for 2 days. Then cells were replaced onto astrocyte coated cover-slips in N2 medium with supplementary factors. The culture medium was changed every day until used.
The morphology changes of the urine cells during this procedure were described here. For the first 4 days, the cells showed epithelial-like morphology and sustained proliferation. From the Day 4, cells began to change their shape. The expression of the transcriptional factors was analyzed on Day 4. We found that almost all the cells expressed GFP, but we could not know which cells expressed the 5 factors at the same time. On the Day 5, about 30% of the cells elongated and became long spindle cells. Some grew dendrite-like structures. Unfortunately, only a small percentage of these cells could be converted into neurons. Most of these cells began to die at almost the same time. Cells which were successfully converted into neurons grew long processes and exhibited neuron-like morphology. And these cells could be labeled by neuron lineage marker Tuj1.
