# Supplementary figures and images for: Modeling Neurological Disease by Rapid Conversion of Human Urine Cells into Functional Neurons
Source: Stem Cells Int. 2015 Dec 7;2016:2452985. doi: 10.1155/2016/2452985 (PMC4685145; doi:10.1155/2016/2452985)

**One**

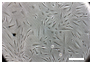

**Three**

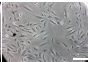

**Five**

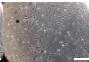

**Seven**

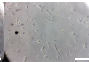

**Nine**

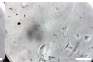

**Eleven**

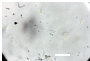

**Thirteen**

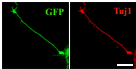

**Four**

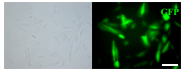

Supplement: Supplementary file 1 — In order to convert urine cells into functional neurons, five retroviruses carrying Ascl1, Brn2, NeuroD, c-Myc, and Myt1l were used. Twenty-four hours after the recovery, the five retroviruses were added to primary urinary cells for one day. The medium was changed to EPi/N2 medium supplied with 1 µg/ml DOX the second day and last for 2 days. Then cells were replaced onto astrocyte coated cover-slips in N2 medium with supplementary factors. The culture medium was changed every day until used. The morphology changes of the urine cells during this procedure were described here. For the first 4 days, the cells showed epithelial-like morphology and sustained proliferation. From the Day 4, cells began to change their shape. The expression of the transcriptional factors was analyzed on Day 4. We found that almost all the cells expressed GFP, but we could not know which cells expressed the 5 factors at the same time. On the Day 5, about 30% of the cells elongated and became long spindle cells. Some grew dendrite-like structures. Unfortunately, only a small percentage of these cells could be converted into neurons. Most of these cells began to die at almost the same time. Cells which were successfully converted into neurons grew long processes and exhibited neuron-like morphology. And these cells could be labeled by neuron lineage marker Tuj1. [file 2452985.f1.pdf]
